# Manipulation of a social signal affects DNA methylation of a stress-related gene in a free-living bird

**DOI:** 10.1242/jeb.246819

**Published:** 2024-07-30

**Authors:** Sabrina M. McNew, Conor C. Taff, Maren N. Vitousek

**Affiliations:** ^1^ Department of Ecology and Evolutionary Biology, Cornell University, Ithaca, NY 14853, USA; ^2^ Cornell Lab of Ornithology, Cornell University, Ithaca, NY 14850, USA; ^3^ Department of Ecology & Evolutionary Biology, University of Arizona, Tucson, AZ 85719, USA; ^4^ Department of Biology, Colby College, Waterville, ME 04901, USA

**Keywords:** *Tachycineta bicolor*, Epigenetic modification, *CRHR1*, *GR*, *FKBP5*, *CRH*

## Abstract

Social status directly affects the health of humans and other animals. Low status individuals receive more antagonistic encounters, have fewer supportive relationships and have worse health outcomes. However, the physiological and cellular processes that mediate the relationship between the social environment and health are incompletely known. Epigenetic regulation of the hypothalamic–pituitary–adrenal (HPA) axis, the neuroendocrine pathway that activates in response to stressors, may be one process that is sensitive to the social environment. Here, we experimentally manipulated plumage, a key social signal in female tree swallows (*Tachycineta bicolor*) and quantified methylation of four genes in the HPA axis before and after treatment. We found that dulling the white breast plumage affected methylation in one gene, *CRHR1*; however, the effect depended on the original brightness of the bird. Methylation in this gene was correlated with baseline corticosterone levels, suggesting that DNA methylation of *CRHR1* helps regulate glucocorticoid production in this species. Methylation in two other genes, *FKBP5* and *GR*, changed over the course of the experiment, independent of treatment. These results show that methylation of these genes is labile into adulthood and suggest that epigenetic regulation of the HPA axis could help birds respond to current environmental conditions.

## Teaser

**Summary:** Experimental manipulation of a social signal in breeding tree swallows reveals epigenetic changes to DNA methylation in one gene in the HPA axis.

## INTRODUCTION

The health of humans and other social animals is affected by their social environment. The social environment includes axes such as social integration, i.e. the ability of individuals to maintain supportive conspecific interactions, as well as social status, i.e. the relative difference among individuals in access to resources or support (Snyder-Mackler et al., 2020). Lower status individuals have shorter lifespans and are more susceptible to disease (Alwin and Wray, 2005; Razzoli et al., 2018; Singh-Manoux et al., 2003). The health effects of social status are partially attributed to variation in access to resources, differences in risk aversion, and other environmental mediators of health (Singh-Manoux et al., 2003; Snyder-Mackler et al., 2019). However, increasing evidence suggests that encounters between individuals living in the same social environment may have direct and lasting physiological consequences (Sapolsky, 2004; Snyder-Mackler et al., 2019, 2020). What remains unclear is what mechanisms govern the effects of social environment on health and fitness.

One link between the social environment and fitness is the hypothalamic–pituitary–adrenal (HPA) axis (Creel et al., 2013). This neuroendocrine pathway underlies physiological and behavioral changes that shift energy towards immediate survival in response to an adverse event (Monaghan, 2014; Sapolsky et al., 2000; Wingfield et al., 1998). Multiple signaling hormones are involved in the pathway and they have complex effects on reproductive, metabolic and immune physiology (MacDougall-Shackleton et al., 2019; Wingfield and Sapolsky, 2003). Particularly important are glucocorticoid hormones, a class of steroid hormones that suppress normal reproductive behavior and immune function while triggering increased foraging and activity, increased gluconeogenesis and other phenotypes associated with an ‘emergency life history stage’ (Sapolsky et al., 2000; Wingfield, 2013; Wingfield et al., 1998). While a short-term increase in glucocorticoid levels is an adaptive response to an acute stressor, long-term activation of the HPA axis and associated chronically high levels of glucocorticoids can have negative effects (Romero et al., 2009; Sapolsky et al., 2000). Thus, the adaptive regulation of these hormones in response to environmental stressors is associated with important survival and fitness outcomes (Bonier et al., 2009; Schoenle et al., 2021; Vitousek et al., 2018, 2019).

Social status can affect glucocorticoid regulation in both positive and negative ways (Creel et al., 2013; Dantzer and Newman, 2022; DeVries et al., 2003). Antagonistic interactions between conspecifics typically raise glucocorticoid levels (Creel et al., 2013; Deviche et al., 2014; Landys et al., 2010). For example, female bluebirds (*Sialia mexicana*) living in dense populations have higher circulating levels of glucocorticoids, which may be transmitted to their eggs, affecting the aggression and dispersal behaviors of their offspring (Potticary and Duckworth, 2020). Low status individuals may receive particularly frequent attacks from conspecifics, leading to negative consequences of chronically elevated glucocorticoid levels (Korte et al., 2005; Snyder-Mackler et al., 2019).

Conversely, social integration and a high social status can also positively affect glucocorticoid regulation. ‘Social buffering’, or the ability of social partners to reduce stress responses, may promote health (Hennessy et al., 2009). Social bonds between primates reduce glucocorticoid levels in the face of stressors (Engh et al., 2006; Young et al., 2014). For instance, male Barbary macaques (*Macaca sylvanus*) with strong social bonds had lower fecal glucocorticoid levels in response to social stressors (aggressive encounters) as well as environmental stressors (cold temperatures). In birds, social buffering has been observed between mates and between mothers and offspring (Edgar et al., 2015; Remage-Healey et al., 2003). Fewer studies in birds have investigated differences in the role of social status in buffering or exacerbating responses to environmental stressors. Rubenstein (2007) found that subordinate superb starlings (*Lamprotornis superbus*) had higher corticosterone during years of environmental stress (low rainfall) than dominant individuals, although it was not clear whether this effect was due to subordinate individuals being lower quality and thus more susceptible to the stress of drought or to supportive social buffering experienced by dominant individuals, or both.

Connections between the social environment and glucocorticoid regulation have thus been well documented; however, the molecular mechanisms that drive and maintain changes to HPA axis activity are unclear. Epigenetic changes to genes involved in the HPA axis could underlie the connection between the social environment and stress-related phenotypes (Lee and Sawa, 2014; Snyder-Mackler et al., 2019; Turecki and Meaney, 2016). Epigenetic modifications, such as DNA methylation, are sensitive to the environment and can affect DNA expression and physiology (Sotnikov and Markt, 2014). A robust body of literature from studies on primates and lab rodents supports the hypothesis that environmental stressors may cause persistent changes in the regulation of genes involved with the stress response (Schartner et al., 2017; Turecki and Meaney, 2016). For instance, one study found that prenatal trauma exposure in mice led to changes in the methylation of two genes in the stress axis, which were accompanied by changes in mRNA levels, corticosterone levels and behavior (Plank et al., 2021). Another study found changes in the social environment of primates can cause changes in chromatin availability and gene expression, leading to dysregulation in the HPA axis and corresponding negative health effects (Snyder-Mackler et al., 2019).

Many bird species are highly social; however, the epigenetic imprint of their social landscape is not well understood. Experimental changes in the social status of house sparrows (*Passer domesticus*) led to increased glucocorticoid levels in larger-bodied birds; however, it is not known whether changes to the methylation of genes in the HPA axis mediated the glucocorticoid response (Lindström et al., 2005). A more recent study found that increased competition in tree swallows (*Tachycineta bicolor*) has effects on DNA methylation and gene expression in brain tissue after just 2 days (Bentz et al., 2021). Effects of increased competition included differential expression in genes such as corticotropin-releasing hormone receptors (*CRHR1*), and glucocorticoid receptors (*GR*) as well as other genes associated with biological processes, including response to hormones and corticosteroid transport.

In this study, we experimentally manipulated a key plumage signal in tree swallows and measured its effects on the methylation of four genes involved in the HPA axis. Plumage is an important social signal in birds, conveying information about condition, parasite load and social dominance (Mason and Bowie, 2020; Mougeot et al., 2010; Taff et al., 2019a). In female tree swallows, brighter white females secrete more corticosterone in response to stress, have stronger immune function and more social interactions with conspecifics, and are less likely to abandon their nests under stressful conditions (Beck et al., 2015; Taff et al., 2019a). Methylation in some regions is correlated with plumage brightness and stress resilience, suggesting that epigenetic processes could connect this plumage signal to physiology (Taff et al., 2019b). Experimental dulling of this plumage alters the social interaction, microbiome and glucose levels of female swallows, and dulled females invest more in reproduction compared with controls (Taff et al., 2021). This shift in behavior and physiology in response to an altered social landscape could be mediated by changes in the methylation of genes in the HPA axis.

We tested whether plumage dulling of female tree swallows changes DNA methylation of genes involved in the HPA axis, and whether DNA methylation of HPA-associated genes is correlated with corticosterone levels. We investigated DNA methylation of four genes: *CRH* (encoding corticotropin releasing hormone), *CRHR1* (encoding corticotropin releasing hormone receptor 1), *FKBP5* (also sometimes referred to as *FKBP51*, encoding FK506 binding protein 51) and *GR* (also called *NR3C1*, encoding glucocorticoid receptor). We targeted these four genes because they all have key roles in the HPA axis, and epigenetic dysregulation of these genes is associated with stress-related phenotypes (Guidotti et al., 2013; Lee and Sawa, 2014; Schartner et al., 2017; Zimmer et al., 2020). Perception of a stressor activates the hypothalamus, which secretes CRH, driving a signaling cascade resulting in the secretion of corticosterone. Decreased methylation of *CRH*, and corresponding increased expression of this gene, are associated with a stress response in mice (Plank et al., 2021). *CRHR1* and *GR* both encode receptors for signaling hormones. Elevated expression of these genes is also associated with stress-related phenotypes in humans and mice, such as depression and panic disorders (Guidotti et al., 2013; Schartner et al., 2017). Finally, *FKBP5* encodes a protein that has an inhibitory effect on *GR* signaling, and which may be a key mediator of HPA axis flexibility (Zimmer et al., 2020, 2021). We predicted that experimental manipulation of tree swallow plumage brightness would cause changes in the methylation of these genes and corresponding changes in corticosterone levels.

## MATERIALS AND METHODS

The work described here was conducted under federal and state scientific collecting permits to MNV (USGS 24,129, USFWS MB42428C; New York State 215 and 2350). All procedures were approved by the Cornell University Institutional Animal Care & Use Board (IACUC protocol 2019-0023 and 2001-0051).

We studied breeding tree swallows in Ithaca, NY, USA, during April to July of 2017 (42°30′11″ N, 76°26′13″ W). Females at each nest were captured 3 times during the breeding season (day 6–7 of incubation, day 3–4 after hatching, and day 7–8 after hatching). At the first capture, females were assigned randomly either to a plumage dulling treatment or to a control treatment after balancing treatments by female age (second year versus after second year). We dulled plumage by uniformly coloring the feathers from the throat to the legs using a light gray non-toxic marker (Faber-Castell PITT Artist Pen ‘Big Brush’ Warm Grey III 272), following methods in Taff et al. (2021). Females in the control treatment were marked in the same way with a colorless marker (Prismacolor Premier Colorless Blender PB-121). The marking treatment was re-applied at the second and third captures. We quantified the effects of dulling through spectrophotometry (see Supplementary Materials and Methods). In total, the dulled group included 34 females and the control group included 36 females. Treatment groups did not differ significantly in initial brightness (average percentage reflectance in the control group 39.85%, dulled group 41.05%; *P*=0.491). Experimental dulling significantly reduced plumage brightness for all individuals in the treatment [mean change in brightness immediately post-treatment −0.99 s.d. units, confidence interval (CI) −0.58, −0.1.39; Taff et al., 2021]. Sham-dulling of females in the control treatment did not affect brightness (mean change in brightness immediately post treatment −0.01 s.d. units, CI −0.47, 0.44).

At the first and third captures (hereafter ‘pre-treatment’ and ‘post-treatment’), we took a small blood sample within 3 min of capture via brachial venipuncture to measure stress physiology and quantify DNA methylation. At the pre-treatment capture, we took two additional blood samples: the first was collected after 30 min to measure maximal corticosterone elevation (‘stress-induced corticosterone’). Immediately after taking the stress-induced sample, we injected birds with 4.5 ml g^−1^ Mylan^®^ (4 mg ml^−1^) dexamethasone sodium phosphate, and then took a final blood sample 30 min later (‘dexamethasone-controlled corticosterone’) (Zimmer et al., 2019). Dexamethasone is a synthetic glucocorticoid that binds to glucocorticoid receptors, reducing the exogenous release of corticosterone by stimulating negative feedback, i.e. down-regulation of HPA activity (Ratka et al., 1989; Zimmer et al., 2019). Within 3 h, erythrocytes and plasma were separated by centrifugation and stored separately at −30°C. Corticosterone was measured in the plasma using commercially available microplate kits that have been validated in this population (Zimmer et al., 2019; Supplementary Materials and Methods). Data on the behavior, microbiome, corticosterone regulation and reproductive success of adults in this experiment have been published previously (Taff et al., 2021). Here, we focused on the effects of plumage manipulation on DNA methylation and their connection to glucocorticoid levels.

We extracted whole genomic DNA from frozen erythrocytes using Qiagen DNEasy Blood and Tissue Kits (Valenica, CA, USA) following the manufacturer's protocol. We assayed DNA concentration and purity on a NanoDrop Spectrophotometer (ThermoFisher Scientific, Waltham, MA, USA) and then shipped purified DNA to EpigenDx (Hopkinton, MA, USA) for methylation quantification. Primer development, assay validation and pyrosequencing were conducted at EpigenDX. Between 23 and 96 primer pairs were designed per gene to assay methylation in each of the four target genes (see Supplementary Materials and Methods for extended details on assay development). Based on initial tests with a separate set of 36 tree swallow samples, a subset of primer pairs with good amplification rates were selected to maximize coverage of regions with high CpG density and high variation in methylation levels. With those criteria, we used three primer pairs to assay methylation in *GR*, and one primer pair each to assay methylation in *CRH*, *CRHR1* and *FKPB5.* Methylation assays targeted 11–19 CpG sites per gene. A total of 121 samples from 70 individual birds were then pyrosequenced. Some birds did not have post-treatment samples either because we failed to recapture them (*N*=15) or because their samples failed extraction (*N*=4).

Pyrosequencing procedures followed standard methods developed by EpigenDx. For each sample, 500 ng of genomic DNA was bisulfite treated using the EZ DNA Methylation kit (Zymo Research, Inc., Irvine, CA, USA). Bisulfite-treated DNA was purified according to the manufacturer's protocol and eluted to a final volume of 46 µl. Then, target regions were amplified in PCR reactions containing 1 µl of bisulfite-treated DNA and 0.2 µmol l^−1^ of each primer. One primer was biotin-labeled and HPLC purified (for subsequent purification with Sepharose beads).

PCR product was bound to Streptavidin Sepharose HP (GE Healthcare Life Sciences), after which the immobilized PCR products were purified, washed, denatured with a 0.2 µmol l^−1^ NaOH solution, and rewashed using the Pyromark Vacuum Prep Tool (Qiagen), as per the manufacturer's protocol. Next, 0.5 µmol l^−1^ of sequencing primer was annealed to the purified single-stranded PCR products and 10 µl of the PCR products was pyrosequenced on the PSQ96 HS System (Qiagen) following the manufacturer's instructions.

The methylation status of each CpG site was determined individually as an artificial C/T SNP using PyroMark software (Qiagen). The methylation level at each CpG site was calculated as the percentage of the methylated alleles divided by the sum of all methylated and unmethylated alleles. Each experiment included non-CpG cytosines as internal controls to detect incomplete bisulfite conversion of the input DNA. In addition, a series of unmethylated and methylated DNA was included as controls in each PCR. Furthermore, PCR bias testing was performed by mixing unmethylated control DNA with *in vitro* methylated DNA at different ratios (0%, 5%, 10%, 25%, 50%, 75% and 100%), followed by bisulfite modification, PCR and pyrosequencing analysis.

### Analysis

We modeled methylation in each gene separately using linear mixed effects models in R (version 4.2.2). Each model predicted per-CpG methylation as a function of capture (pre- or post-treatment), treatment and initial plumage brightness. We first created an interaction model of the three main effects (i.e. capture×brightness×treatment). When interactions were not significant, they were removed, and we present estimates from the additive model instead. Models also included the random effect of CpG site and the random effect of individual. We logit transformed methylation data prior to modeling it following best-practices for percentage data (Stevens et al., 2016; Warton and Hui, 2011). We partitioned the variance in methylation among the fixed effects and random effects using rptR (https://CRAN.R-project.org/package=rptR) to compare how much variation in methylation was explained by experimental variables versus between-individual differences (Stoffel et al., 2017). Second, we tested for a relationship between corticosterone levels and methylation at each gene by modeling methylation as a function of corticosterone, using methylation and corticosterone data from both captures. These models included the random effects of individual and CpG identity. Conceptually, we predicted that DNA methylation controls gene expression in the HPA axis and thus affects blood corticosterone levels (i.e. corticosterone depends on DNA methylation). However, because of the hierarchical structure of the data, we found it more appropriate to model DNA methylation as the dependent variable (with corticosterone as a fixed effect and CpG as a random effect).

## RESULTS

We quantified methylation data at 56 CpG sites in the four focal genes (*CRH*, *FKBP5*, *GR* and *GRHR1*). We obtained methylation data from between 85 and 120 samples at each CpG (Dataset 1). The mean methylation per site varied from 2.1% to 73.1%.

**Fig. 1.**
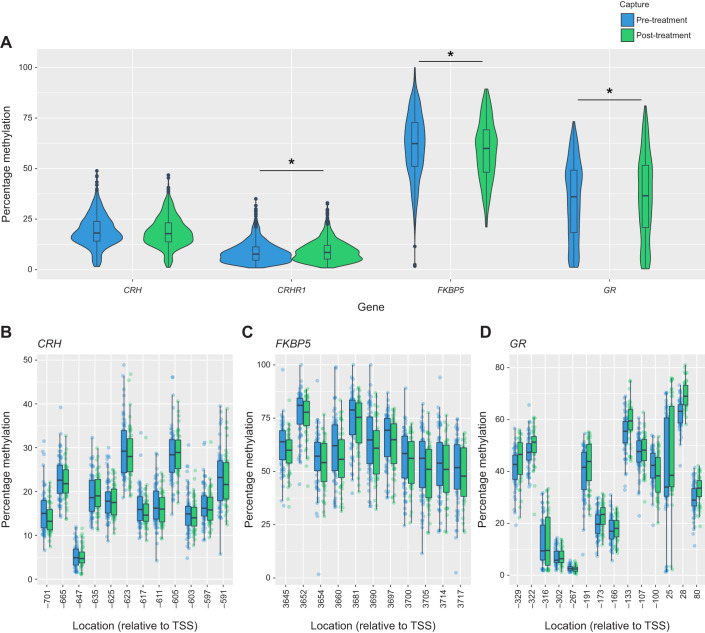
**Quantification of methylation in the four focal genes: *CRH*, *FKBP5*, *GR* and *GRHR1*.** (A) Distribution of percentage methylation across all interrogated CpG sites for the four genes. Asterisks indicate significant differences in methylation between the two sampling points. (B–D) Percentage methylation at CpG sites in *CRH*, *FKBP5* and *GR*, respectively. The location of CpG sites is given relative to the transcription start site (TSS). Box plots show median, upper and lower quartiles and 1.5× interquartile range.

**Fig. 2.**
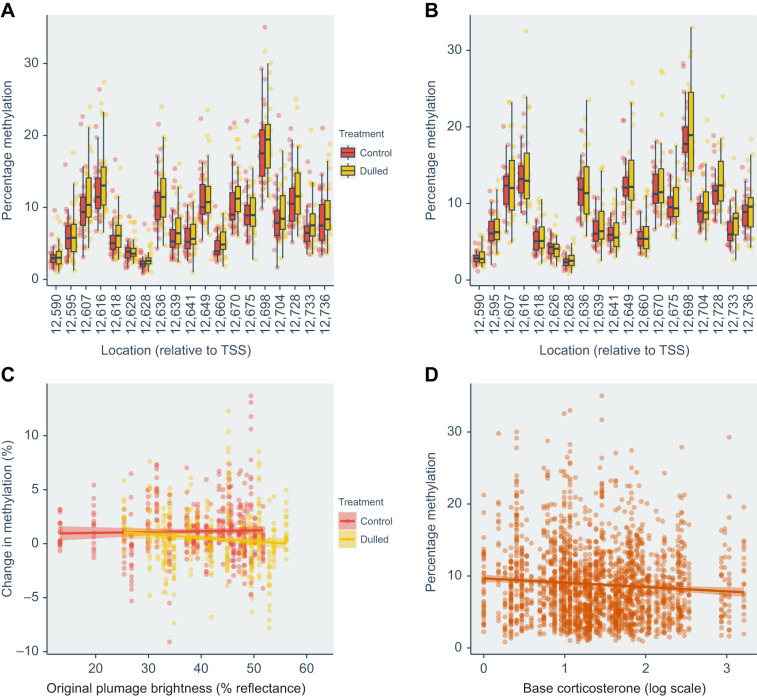
**Percentage methylation at CpG sites in *CRHR1* before and after plumage dulling.** (A) Pre-manipulation. (B) Approximately 14 days post-manipulation. (C) There was a significant interaction between the original plumage brightness of individual birds and the effect of treatment on methylation in *CRHR1* (LMM *P*=0.02, Conditional *R*^2^=0.884). (D) Baseline corticosterone and percentage methylation of *CRHR1* were significantly negatively correlated (LMM *P*<0.001, Conditional *R*^2^=0.877). Points in D include data from both pre- and post-treatment captures. Lines in C and D represent linear model regression lines for each group. The shaded area depicts the 95% confidence interval for the estimate.

Methylation of the *CRH* gene did not significantly differ between treatments or between captures and was not associated with initial female brightness (Fig. 1A,B, Table 1). Methylation in the *FKBP5* gene was significantly higher pre-treatment compared with post-treatment (Fig. 1A,C, Table 1). However, there was no significant difference between treatments, and no relationship between initial brightness and methylation. In contrast, methylation in the *GR* gene was significantly lower pre-treatment compared with post-treatment (Fig. 1A,D, Table 1). Again, methylation did not significantly differ between treatments and was uncorrelated with initial brightness. Finally, we found that methylation of the *CRHR1* gene depended on the three-way interaction between treatment, capture and brightness (Fig. 2A–C, Table 1). Females in the dulled treatment tended to decrease DNA methylation levels in the *CRHR1* gene following experimental dulling. The decrease in methylation post-treatment was strongest for females that were originally bright. In contrast, for females in the control group, methylation tended to increase slightly post-treatment and did not depend on initial brightness.

**Table 1.**
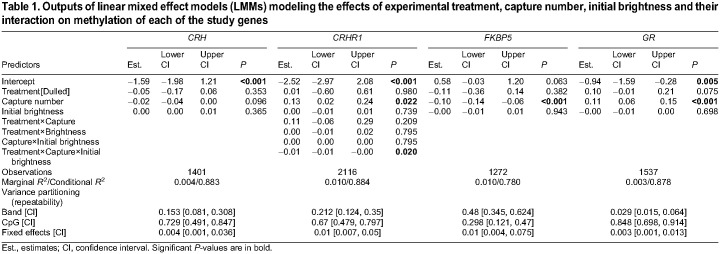
Outputs of linear mixed effect models (LMMs) modeling the effects of experimental treatment, capture number, initial brightness and their interaction on methylation of each of the study genes

In all genes, the random effects of individual and CpG explained substantially more variation than did the fixed effects of treatment, capture number and initial brightness (i.e. Conditional *R*^2^>>Marginal *R*^2^; Table 1). Individual bird identity alone explained between 2.9% and 48% of variation in methylation at each gene (Table 1).

Methylation of *CRHR1* was significantly associated with baseline corticosterone levels. There was a significant negative relationship between baseline corticosterone and methylation in *CRHR1* (LMM *P*<0.001; Fig. 2D; Table S1). Baseline corticosterone was not associated with methylation of any other of the three genes (Table S1). There was no significant association between pre-treatment stress-induced corticosterone and methylation of any of the four genes (Table S2). There was also no significant association between pre-treatment dexamethasone-controlled corticosterone and methylation in any of the four genes (Table S3). Stress-induced and dexamethasone-controlled corticosterone were only quantified pre-treatment, so we did not test for an effect of treatment on those measurements. Baseline corticosterone did not differ between dulling and control groups post-treatment (Taff et al., 2021).

## DISCUSSION

In this study, we tested the effects of manipulating a key social plumage signal of tree swallows on methylation of four genes involved in the HPA axis. We found that experimentally dulling the white breast plumage resulted in changes in the methylation of the *CRHR1* gene. The effect of dulling on methylation was strongest for females that were initially bright, suggesting that high-status females experienced the strongest consequences of social environment change. CRHR1 binds CRH, triggering the release of the adrenocorticotropic hormone, which leads to the release of corticosteroids (Schartner et al., 2017). Decreased methylation of *CRHR1* and associated upregulation of this gene are associated with anxiety-related phenotypes in humans and rodent models (Plank et al., 2021; Schartner et al., 2017; Sotnikov and Markt, 2014). Baseline corticosterone values in our tree swallows were negatively associated with methylation across this gene. Thus, the decrease in methylation that we observed in females is consistent with the upregulation of this gene and activation of glucocorticoid hormones in response to plumage dulling and the concomitant changes to the social environment (Taff et al., 2021).

In contrast, the methylation of the other three genes we studied (*GR*, *FKBP5* and *CRH*) was not significantly associated with treatment. We chose these candidate genes as targets because they have known epigenetic associations with stress in humans and model organisms (Lee and Sawa, 2014). Still, we interrogated relatively few sites across these specific genes and so it is possible that we did not detect some of the methylation effects of the plumage manipulation. The locations of CpGs within the genes may also differ in their sensitivity to environmental stressors. All the sites we interrogated in *CRHR1* and *FKBP5* were in the gene body, whereas CpGs in *CRH* and *GR* were mostly in the promoter region (Dataset 1). The stability and also the functional effects of DNA methylation likely differ between CpGs in the gene body versus promoter region (Derks et al., 2016; Lindner et al., 2021). We preferentially selected a subset of CpGs in our candidate genes during assay development, prioritizing those sites with good amplification and variability in percentage methylation among individuals. However, deeper sequencing of a larger set of loci would provide additional resolution of the mechanisms linking environmental stressors, the HPA axis and physiological phenotype.

In addition, our use of erythrocytes as a sample tissue may have limited our ability to detect methylation changes. DNA methylation differs among cell and tissue types and methylation changes in response to environmental stress may have been limited to specific areas, such as brain or adrenal tissue (Bentz et al., 2021; Plank et al., 2021; Turecki and Meaney, 2016). Nevertheless, other studies have found that blood can be a useful, albeit imperfect, proxy for studying epigenetic responses of wild birds to environmental stressors over short to medium time scales (Husby, 2020; Lindner et al., 2021; McNew et al., 2017). In our study, using blood allowed us to non-destructively sample birds pre- and post-treatment, increasing our power to detect within-individual changes in methylation.

Previous analyses of the physiological and behavioral effects of this experiment found that plumage dulling altered social interactions and changed microbiome diversity and glucose levels of female birds (Taff et al., 2021). However, there was no significant effect of treatment on corticosterone levels. Thus, the fact that we similarly saw no treatment effect on methylation in three of the four genes we studied suggests that plumage manipulation had relatively minor effects on aspects of the HPA axis that regulate glucocorticoids. Previous analysis of behavioral changes in response to plumage dulling indicated that manipulation of this signal affects the social landscape in subtle and complex ways. Indeed, many of the effects of treatment identified by Taff et al. (2021) were dependent on nestling stage and initial female brightness. More work is needed to understand exactly how manipulation of white plumage in tree swallows affects their social environment.

The methylation of two genes (*GR* and *FKBP5*) was not affected by treatment; however, methylation did change significantly between the two time points in the study (pre-treatment: days 6–7 of incubation; and post-treatment: ∼14 days later). Our data thus add to a growing body of literature demonstrating that DNA methylation can be dynamic over short time scales, even in adult animals, and may be sensitive to environmental cues and stressors (Mäkinen et al., 2019; Viitaniemi et al., 2019). Environmental factors besides our experimental treatment could have affected methylation of these genes over the course of the experiment.

The glucocorticoid receptor (GR, also called NR3C1) is an intracellular transcription factor that mediates the expression of several proteins involved in the stress response (Guidotti et al., 2013; Zannas et al., 2016). Methylation of *GR* increased over the study period in our swallows, which could have resulted in reduced expression of this gene. This change could have been part of a modulation of the HPA axis during breeding, which may be important because HPA activity can inhibit reproductive success (Bókony et al., 2009; Wingfield and Sapolsky, 2003). Alternatively, methylation of this gene may also be related to environmental conditions. A previous study of superb starlings (*L. superbus*) found that methylation in the promoter of *GR* was positively correlated with environmental conditions (rainfall) early in life (Rubenstein et al., 2016). In that study, epigenetic programming of the *GR* gene early in life was suggested to underlie adaptive plasticity of stress phenotypes as adults living in highly variable environments. The change that we observed over our study shows that methylation of this gene is labile over relatively short periods (∼2 weeks) in adults. Thus, methylation in this gene may help birds adapt to variable environments both during development and into adulthood.

The other gene that differed between time points in our study, *FKBP5*, is a negative regulator of *GR* signaling (Menke et al., 2013; Zannas and Binder, 2014). Dysregulation of *FKBP5* expression is associated with psychiatric disorders and other stress-related phenotypes in humans and laboratory models (Zannas et al., 2016; Zimmer et al., 2020). Although data from wild organisms are limited, *FKBP5* expression in house sparrows (*Passer domesticus*) is correlated with HPA flexibility and exploratory behavior (Zimmer et al., 2021). *FKBP5* is thus emerging as a key regulator of the HPA axis across many vertebrates (Zimmer et al., 2020). In our study, methylation of *FKBP5* decreased over the course of the study period. Decreased methylation of *FKBP5* is expected to upregulate this gene and inhibit glucocorticoid receptor signaling (Zannas et al., 2016). Correspondingly, the change that we observed in our birds could also be related to an effort to downregulate the stress response during breeding. *FKBP5* was also notable because individual bird identity explained a substantial proportion (48%) of the variation in methylation in this gene (Table 1). The consistency in methylation of *FKBP5* across individuals suggests that perhaps some epigenetic programming of this gene occurs early in life and/or is transgenerationally inherited. In fact, another study found that methylation of cytosines in *FKBP5* is a heritable epigenetic marker of trauma in humans (Yehuda et al., 2016), supporting the idea that *FKBP5* could mediate physiological effects of both current and historical stressors.

A growing number of studies have investigated the plasticity of DNA methylation in response to environmental conditions in free-living animals. However, most of these studies have focused on early life methylation programming (McNew et al., 2021; Rubenstein et al., 2016; Sheldon et al., 2020; Watson et al., 2019) and studies that sample the same individuals repeatedly are particularly rare (e.g. Anderson et al., 2021; Rubenstein et al., 2016). Our results highlight that DNA methylation patterns can change within an individual in response to specific experimental manipulation and, moreover, may be naturally labile over short periods. Although we did not see changes across all the genes we studied in response to plumage dulling, the change in methylation in *CRHR1* suggests that epigenetic mechanisms may mediate the effects of social environment on the physiology of free-living social birds.

## Supplementary Material

10.1242/jexbio.246819_sup1 Supplementary information

Dataset 1. Summary data for each of the CpGs characterized in this study including minimum, median, mean, and maximum methylation, and the number of samples sequenced at that site
