# Supplementary material for: Manipulation of a social signal affects DNA methylation of a stress-related gene in a free-living bird
Source: J Exp Biol. 2024 Jul 30;227(15):jeb246819. doi: 10.1242/jeb.246819 (PMC11418189; doi:10.1242/jeb.246819)
Supplement: Supplementary information [file jexbio-227-246819-s1.pdf]

Supplementary Materials and Methods

Plumage measurement

The coloration of female breast plumage was quantified following methods developed previously in this system [1,2]. We collected feathers from the center of the breast at each capture to quantify initial female brightness. Reflectance was measured with an Ocean Optics FLAMES-UV-VIS spectrophotometer with PX-2 pulsed Xenon light source and WS-1 white standard in OceanView v.1.5.2 (Ocean Optics, Dunedin, FL, U.S.A.). For each individual sample, we stacked four feathers on an index card and taped them in place before smoothing the barbs. We used a fibre-optic UV/VIS probe positioned in a holster that blocked external light and measured spectra at a fixed distance of 5 mm from the feather sample. Then, we collected and saved four separate spectra for each feather stack; each spectra had a 10 scan average, 20 nm boxcar width and 60 ms integration time. The probe was removed from the feather patch and replaced between measurements. For each female in the study, we measured four sets of feathers (two from the first capture and one each from the second and third captures). Raw reflectance spectra generated by OceanView were processed in R v.3.3.3 (R Core Team, 2016) using the package 'pavo' (Maia, Eliason, Bitton, Doucet, & Shawkey, 2013). We calculated mean total breast brightness as the average reflectance from 300 to 700 nm ('B2' in the 'pavo' package). We then averaged the four repeated measurements from each feather sample to calculate a final brightness measure.

Corticosterone quantification

We measured baseline, stress-induced, and post-dexamethasone corticosterone levels in blood plasma samples using commercially available enzyme immunoassay (EIA) kits (DetectX Corticosterone, K014eH5, Arbor Assays, Ann Arbor, MI, U.S.A.) that were previously validated for use in tree swallows (Taff, Zimmer et al., 2019). Briefly, corticosterone was extracted from 5 μl of plasma using a triple ethyl acetate extraction. Samples were then run in duplicate following the EIA kit manufacturer's protocol. Extraction efficiency was assessed by spiking samples with a known amount of corticosterone; average extraction efficiency was 89.7%. The lower detection limit was 0.8 ng/ml. Inter-assay variation was 5.7%, and intra-assay variation was 10.6%.

Methylation Assay Development

Initially, we sent a set of 36 tree swallow samples (that were not part of the experiment reported in this study) to develop assays for targeted methylation analysis of our four candidate genes (GR, CRHR1, FKBP5, and CRH). For these 36 samples, EpigenDx used targeted next generation bisulfite sequencing (tNGBS) to assay methylation percentages for CpGs near each of the genes with a focus on coverage of CpG rich regions immediately upstream of the TSS and in the exons of each gene body. Sequencing was accomplished by scaffolding primer pairs along the gene and flanking regions. Using this approach, we received data for a total of 145 CpGs for GR from 23 primer pairs, 171 CpGs for CrH from 24 primer pairs, 130 genes for FKBP5 from 96 primer pairs, and 67 CpGs for CRHR1 from 96 primer pairs.

We used the tNGBS data from these 36 samples to select a smaller subset of primer pairs to pursue with pyrosequencing in the experiment reported here. To make this selection, we first excluded primer pairs in which the CpGs had very low between-individual variation (usually cases in which all CpGs were near 0 or 100% methylation). We also excluded pairs that yielded data on only a small

number of CpGs or that failed to amplify consistently. In sum, we focused our targets to areas of the genome that appeared to have high between-individual variation and good amplification rates. Using those criteria, we selected three primer pairs in GR and one primer pair each in CRH, CRHR1, and FKBP5 to target for pyrosequencing in our experiment. We sent a total of 121 samples from 70 individual birds to EpigenDx for pyrosequencing to quantify methylation in the selected areas of the four target genes.

References

1. Taff CC, Zimmer C, Scheck D, Ryan TA, Houtz JL, Smee MR, Hendry TA, Vitousek MN. 2021 Plumage manipulation alters associations between behaviour, physiology, the internal microbiome and fitness. *Animal Behaviour* **178**, 11–36. (doi:10.1016/j.anbehav.2021.05.012)

2. Taff CC, Zimmer C, Vitousek MN. 2019 Achromatic plumage brightness predicts stress resilience and social interactions in tree swallows (Tachycineta bicolor). *Behavioral Ecology* **30**, 733–745. (doi:10.1093/beheco/arz010)

Table S1. LMM output modeling the relationship between baseline corticosterone and methylation of the focal genes.

	CRH			CRHR1			FKBP5			GR		
Predictors	*Estimates*	*CI*	*p*	*Estimates*	*CI*	*p*	*Estimates*	*CI*	*p*	*Estimates*	*CI*	*p*
Intercept	-1.52	-1.81 – -1.22	**<0.001**	-2.34	-2.57 – -2.10	**<0.001**	0.46	0.19 – 0.74	**0.001**	-0.89	-1.49 – -0.28	**0.004**
Baseline corticosterone	0.00	-0.00 – 0.00	0.967	-0.01	-0.01 – -0.00	**<0.001**	0.00	-0.00 – 0.01	0.251	-0.00	-0.01 – 0.01	0.893
**Random Effects**												
σ^2	0.04			0.05			0.13			0.19		
τ_{00}	0.05 Band			0.08 Band			0.27 Band			0.05 Band		
	0.26 cpg			0.25 cpg			0.17 cpg			1.32 cpg		
ICC	0.88			0.88			0.78			0.88		
N	12 cpg			19 cpg			11 cpg			14 cpg		
	69 Band			69 Band			70 Band			69 Band		
Observations	1412			2129			1283			1551		
Marginal R^2 / Conditional R^2	0.000 / 0.882			0.003 / 0.877			0.001 / 0.775			0.000 / 0.877		

Journal of Experimental Biology • Supplementary information

Table S2. LMM output modeling the relationship between stress-induced corticosterone and methylation of the focal genes.

	CRH			CRHR1			FKBP5			GR		
Predictors	*Estimates*	*CI*	*p*	*Estimates*	*CI*	*p*	*Estimates*	*CI*	*p*	*Estimates*	*CI*	*p*
Intercept	-1.40	-1.72 – -1.08	**<0.001**	-2.41	-2.69 – -2.13	**<0.001**	0.53	0.13 – 0.93	**0.009**	-0.86	-1.47 – -0.26	**0.005**
Stress-induced cort	-0.00	-0.01 – 0.00	0.087	-0.00	-0.01 – 0.01	0.959	-0.00	-0.01 – 0.01	0.940	-0.00	-0.01 – 0.00	0.242
**Random Effects**												
σ^2	0.04			0.05			0.18			0.21		
τ_{00}	0.06 $_{Band}$			0.09 $_{Band}$			0.29 $_{Band}$			0.04 $_{Band}$		
	0.26 $_{cpg}$			0.24 $_{cpg}$			0.18 $_{cpg}$			1.25 $_{cpg}$		
ICC	0.87			0.88			0.72			0.86		
N	12 $_{cpg}$			19 $_{cpg}$			11 $_{cpg}$			14 $_{cpg}$		
	68 $_{Band}$			68 $_{Band}$			69 $_{Band}$			68 $_{Band}$		
Observations	810			1213			757			880		
Marginal R^2 / Conditional R^2	0.007 / 0.876			0.000 / 0.876			0.000 / 0.716			0.001 / 0.863		

Journal of Experimental Biology • Supplementary information

Table S3. LMM output modeling the relationship between corticosterone after dexamethasone induction (to induce negative feedback) and methylation of the focal genes.

	CRH			CRHR1			FKBP5			GR		
Predictors	*Estimates*	*CI*	*p*	*Estimates*	*CI*	*p*	*Estimates*	*CI*	*p*	*Estimates*	*CI*	*p*
Intercept	-1.48	-1.77 – -1.18	**<0.001**	-2.39	-2.63 – -2.15	**<0.001**	0.56	0.27 – 0.86	**<0.001**	-0.96	-1.55 – -0.37	**0.001**
Post-dex corticosterone	-0.00	-0.00 – 0.00	0.155	-0.00	-0.00 – 0.00	0.387	-0.00	-0.01 – 0.00	0.351	0.00	-0.00 – 0.00	0.164
**Random Effects**												
σ^2	0.04			0.05			0.18			0.20		
τ_{00}	0.06 Band			0.09 Band			0.28 Band			0.04 Band		
	0.26 cpg			0.24 cpg			0.18 cpg			1.24 cpg		
ICC	0.88			0.88			0.72			0.86		
N	12 cpg			19 cpg			11 cpg			14 cpg		
	69 Band			69 Band			70 Band			69 Band		
Observations	822			1232			768			894		
Marginal R^2 / Conditional R^2	0.005 / 0.876			0.003 / 0.877			0.006 / 0.717					

Dataset 1. Summary data for each of the CpGs characterized in this study including minimum, median, mean, and maximum methylation, and the number of samples sequenced at that site

Available for download at
https://journals.biologists.com/jeb/article-lookup/doi/10.1242/jeb.246819#supplementary-data
